# Gasdermin: a novel therapeutic target for tumour treatment by activating anti-tumour immunity

**DOI:** 10.1038/s41392-020-0180-4

**Published:** 2020-05-13

**Authors:** Caiyun Fu

**Affiliations:** Zhejiang Provincial Key Laboratory of Silkworm Bioreactor and Biomedicine, College of Life Sciences and Medicine, Zhejiang Sci-Tech University, Hangzhou, 310018 China

**Keywords:** Cancer therapy, Tumour immunology

**Fig. 1:**
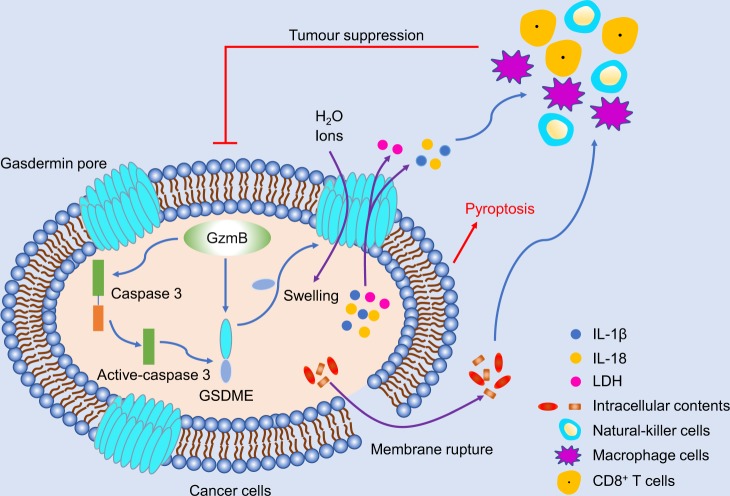
GSDME cleavage by GzmB/caspase 3 promoted pore formation to induce pyroptosis and suppress tumour growth through the enhancement of anti-tumour adaptive immunity

**A very recent study by Zhang et al. published in*Nature*demonstrates that gasdermin E (GSDME, also known as DFNA5) is a tumour suppressor by activating caspase-independent pyroptosis to enhance anti-tumour immunity.** ^1^**In the meantime, a study by Wang et al. published in*Nature*also demonstrates the anti-tumour effect caused by gasdermin A3 (GSDMA3) to induce pyroptosis requires both cytotoxic T cells and CD4** ^+^**T helper cells.** ^2^**The authors intriguingly showed that pyroptosis-induced inflammation triggers robust anti-tumour immunity, thus gasdermin is a promising novel therapeutic target for tumour treatment (Fig.** **1****).**

Pyroptosis is one kind of programmed necrosis mediated by the gasdermin family.^3^ The gasdermin family includes GSDMD, GSDMA, GSDMB, GSDMC, GSDME, and DFNB59. GSDMD is identified as the pyroptotic substrate of caspase-1/4/5/11 and multiple gasdermin-N domains can bind membrane lipids and bear a membrane pore-forming activity with the inner diameters of 10–14 nm of most gasdermin pores.^4^ The gasdermin pores disrupt the balance of the cell membrane to induce water inflow and cell swelling, as well as the release of many proinflammatory cytokines.^5^ GSDME can be cleaved by caspase 3 to switch apoptosis to pyroptosis. Although the expression level of GSDME is suppressed with the potential as a tumour suppressor, it is still unclear whether and how GSDME acts as a tumour suppressor.

The Judy Lieberman group demonstrated that GSDME function was reduced in 20 of 22 tested cancer-associated GSDME mutation.^1^ Zhang et al. addressed the issue of GSDME function in tumour treatment in vitro and in vivo. GSDME function of tumour suppression was mediated by activating caspase-independent pyroptosis, which depended on an involvement of the enhancement of phagocytosis of tumour cells by tumour-associated macrophages, as well as the number and functions of tumour-infiltrating natural-killer and CD8^+^ T lymphocytes.^1^

Using The Cancer Genome Atlas database, the authors first analysed the expression levels of *Gsdme* messenger RNA and/or protein in primary breast cancers and colorectal cancer to seek the tumour model with GSDME high or poor expression level. *Gsdme* was knocked out in highly expressing cell lines (EMT6, CT26, and SH-SY5Y), or stably expressed in poorly expressing cell lines (B16-F10, 4T1E, and HeLa). The tumours knocked out for *Gsdme* in highly expressing cells grew much faster accompanied by fewer CD8^+^ T and NK cells, as well as tumour-associated macrophages. Meanwhile, tumour-infiltrating lymphocytes (TILs: CD8^+^ T and NK cells) from *Gsdme*^−/−^ tumours also expressed less granzyme B (GzmB), perforin (PFN), interferon-γ (IFNγ), and TFN. The overexpression of *Gsdme* in cells with poorly expressing of *Gsdme* had the opposite effect. These results indicated that GSDME has the function of tumour suppression and TIL promotion.

Next, the authors wanted to know whether the tumour suppression of GSDME was mediated by enhanced immune function. The authors provided further evidence that GSDME-mediated tumour inhibition was reduced significantly in mice lacking either CD8^+^ T or NK cells, indicating that killer lymphocytes mediate tumour suppression of GSDME. To determine whether killer lymphocytes induced pyroptosis, the authors used human NK line YT or NK-92 incubated with empty-vector and hGSDME-overexpressing HeLa cells. The results showed that NK cells induced pyroptosis in caspase-dependent and caspase-independent manners. Moreover, the authors used ethylene glycoltetraacetic acid (EGTA) to inhibit cytotoxic granule release and PFN with the results that EGTA completely blocked pyroptosis. All these results indicated that pyroptosis activated by killer cells depended on cytotoxic granule release.

Furthermore, the authors speculated that Gzm proteases were involved in NK-induced pyroptosis by cleavage of GSDME. Based on the results of co-incubation GzmB and GSDME or GSDME D270A mutant, the authors determine that GzmB cleaved GSDME at D270, the same site of caspase 3. Then, the authors hypothesised that mutation of this residue in tumours should abrogate tumour suppression. B16 and 4T1E cells were used to test this hypothesis. The results showed that only tumours overexpressing wild-type GSDME reduced tumour growth, while tumours overexpressing D270A GSDME or empty vector grew with no difference. The authors provided further evidence that the function of killer lymphocytes (CD8^+^ T or NK cells) was enhanced in tumours with the overexpression of wild-type GSDME. All these results provided evidence that cleavage of GSDME at D270 to disturb cell membranes via pore formation was required for tumour suppression.

Altogether, the study by Zhang and colleagues^1^ elegantly illustrates how GSDME acted as a tumour suppressor by inducing pyroptosis in melanoma, triple-negative breast cancer, and colorectal cancer tumours. The enhancement of anti-tumour killer-cell cytotoxicity was necessary and essential for tumour inhibition of GSDME. GSDME cleavage at D270 by GzmB/caspase 3 promoted pore formation to induce pyroptosis and suppress tumour growth through the enhancement of anti-tumour adaptive immunity.

In the meantime, Feng Shao and Zhibo Liu groups^2^ established a bioorthogonal chemical system to enable the controlled release of a drug from an antibody–drug conjugate in mice. Using this bioorthogonal system, the authors observed membrane enrichment of gasdermin N domains after GSDMA3 conjugated to 60-nm nanoparticles and pyroptotic morphology, which phenomenon was deficient when GSDMA3 was mutant with E14K and L184D. Furthermore, the tumour regression induced by GSDMA3 conjugated to nanoparticles was inhibited by IL-1β antibody markedly. The authors also demonstrated that treatment with GSDMA3 conjugated to nanoparticles could synergize with checkpoint blockade anti-PD1 to prevent tumour growth. All these results indicated that pyroptosis induced by GSDMA3 could trigger robust antitumour immunity and GSDMA3 was a novel target for tumour treatment.

In a word, the two papers recently both published in *Nature*^1,2^ gave us novel insights that gasdermin N domains could enrich on the cell membrane to form pores and induce pyroptosis. Antitumour immunity was triggered by inflammation induced by pyroptosis, which was essential for tumour suppressor of gasdermin. Thus, it is a promising way to develop therapeutic strategies depending on gasdermin function, such as the use of the DNA methylation inhibitor decitabine, or combined therapeutics of gasdermin and checkpoint blockade, in order to eradicate tumours by eventually activating robust antitumour immunity.
